# The Severity of Retinopathy in the Extremely Premature Infants

**DOI:** 10.1155/2017/4781279

**Published:** 2017-10-18

**Authors:** Alexandra Trivli, Maria Polychronaki, Charoula Matalliotaki, Michail Papadimas, Athina E. Patelarou, Niki Dermitzaki, Michail Matalliotakis

**Affiliations:** ^1^Department of Ophthalmology, Konstantopouleio General Hospital, Athens, Greece; ^2^Department of Obstetrics and Gynecology, Venizeleio General Hospital of Heraklion, Crete, Greece; ^3^Neonatology Department, Venizeleio General Hospital of Heraklion, Crete, Greece; ^4^Department of Anesthesiology, University Hospital of Heraklion, Crete, Greece

## Abstract

**Objective:**

We aimed to investigate the incidence and the severity of retinopathy of extremely premature infants and to evaluate the risk factors and outcome of the cases.

**Materials and Methods:**

Out of 200 premature births, we retrospectively reviewed 9 cases that developed ROP. We excluded cases where ROP developed in newborns > 30 weeks of gestational age and cases where medical notes were unavailable or incomplete. Topical drops of cyclopentolate 1% and phenylephrine 5% were instilled and fundoscopy was performed using a direct ophthalmoscope.

**Results:**

The incidence of ROP was 4.5% in the 9-year period. The infants were divided into two groups. Group 1 included premature infants ≤27 weeks of age and Group 2 included those >27 weeks but ≤ 30 weeks of age. We found that the infants of Group 1 showed advanced stages of ROP in comparison to Group 2. Out of 18 eyes, 11 eyes had stage 3 ROP and they were all found in Group 1 (100% of cases).

**Conclusion:**

The severity of ROP was associated with earlier gestational age, lower birth weight, and oxygen supplementation. Constant cooperation between physicians and nursing staff is necessary to avoid undetected cases and further prevent ROP related blindness.

## 1. Introduction

Retinopathy of prematurity (ROP) is a major cause of preventable blindness in children, despite current laser or surgical treatment [1].

ROP is estimated to account for 6% to 18% of childhood blindness in developed countries with an infant mortality rate of less than 10 per 1,000 live births [2].

It was first described by Terry in 1942 as retrolental fibroplasia [1]. ROP was initially associated with premature birth and shortly after with oxygen treatment.

It is characterized by abnormal neovascularization of the retina of premature infants [3].

Although laser ablation treatment has been estimated to reduce the incidence of blindness by 25% in the late stages, children still have poor visual acuity after the treatment [1].

In the framework of this study, we aim to estimate the prevalence of ROP in preterm infants and further to evaluate the risk factors and outcome of these cases.

## 2. Materials and Methods

This retrospective study was conducted in the Obstetrics and Gynecology and Neonatology Departments of Venizeleio General Hospital of Heraklion of Crete and involved 200 premature infants, between 2008 and 2017.

ROP was staged according to the International Classification of Retinopathy of Prematurity (ICROP) [4].

A detailed history included the gender, the gestational age, the birth weight, the need for intubation and blood transfusion, and the appearance of sepsis. Topical drops of cyclopentolate 1% and phenylephrine 5% were instilled and fundoscopy was performed after one hour using a direct ophthalmoscope. In our inclusion criteria, we selected 7 singleton premature infants and one twin case. The primary exclusion criteria were cases where ROP developed in newborns of more than 30 weeks of gestational age and the cases where medical notes were unavailable or incomplete.

Additionally, we further evaluated the risk factors that contribute to the appearance of ROP.

*t*-test was used for comparison of the means of the various characteristics and outcome measures. The results were reported as mean ± SD or as percentages where appropriate.

## 3. Results

In total, 9 cases (18 eyes) were recruited in this study. The incidence of developing ROP was 4% in the 9-year period. The infants were divided into two groups based on their gestational age.

Group 1 included infants ≤27 weeks of age (*n* = 6 cases), and Group 2 included infants > 27 weeks but ≤ 30 weeks of age (*n* = 3 cases).

In Group 1, mean gestational age was 25.8 weeks ± 1.3 and mean birth weight was 880 ± 180 gr. Intubation time ranged from 7 to 138 days, 5 infants needed blood transfusions, and 2 infants developed sepsis. Out of 12 eyes in Group 1, one eye developed stage 2 ROP, one eye developed mild stage 3 ROP, two eyes had moderate stage 3 ROP, and 8 eyes had severe stage 3 ROP, and laser photocoagulation treatment was performed in a specialized center.

In Group 2, mean gestational age was 29 weeks ± 1 and mean birth weight was 1120 ± 200 gr. Intubation time ranged from 1 to 49 days, and 2 infants needed blood transfusions. One infant developed stage 1 ROP in both eyes and the other two had stage 2 ROP in both eyes, respectively (Table 1).

In all cases, we noticed respiratory distress syndrome (RDS) and the subsequent need for oxygen support (Table 2).

Finally, we observed that the infants of the first group showed advanced stages of ROP in comparison to the second group. Out of 18 eyes, 11 eyes had stage 3 ROP and they are all found in Group 1 (100% of cases), while 5 eyes had stage 2 ROP (20% in Group 1 and 80% in Group 2) and 2 eyes had stage 1 ROP, both found in Group 2.

## 4. Discussion

ROP is a multifactorial disorder that affects the normal retinal vessel development in premature infants. It is a biphasic disease with an initial phase of vessel growth retardation, followed by a second phase of vessel proliferation. The first phase occurs from birth to postmenstrual age 30–32 weeks. At this time, the nonvascularized retina becomes metabolically active, leading to tissue hypoxia. This hypoxia induces neovascularization by the upregulation of angiogenic growth factors such as vascular endothelial growth factor (VEGF) and erythropoietin. This second phase begins at 32–34-week postmenstrual age [3].

Despite current laser treatment, as well as research for potential new treatments, it remains a significant cause of childhood blindness.

The increase of the survival rates of the extremely premature infants, due to the advancement of neonatal care, has led to an increase in the prevalence of ROP.

According to the literature, the major risk factors for ROP are oxygen use and gestational age/birth weight and have been identified shortly after the initial description of the disease [5, 6].

In utero, the blood is approximately 70% saturated and PaO_2_ is 30 mmHg, while in room air it is 100% saturated and PaO_2_ is 60–100 mmHg [7]. Our data report the existence of RDS in all cases. This can be explained due to the early gestational age; however, this condition raises the need for oxygen supplementation for an extended period of time.

Several studies suggested that although there was a clear connection between ROP and prolonged oxygen use (>7 days), there was no relation to the stage of the ROP, which is also consistent in our case [8].

Regarding the association of early gestational age and ROP, our data support that it is the most significant risk factor, a finding that agrees with the results of other reports [9, 10]. Moreover, it is postulated in the literature that infants with gestational age less than 28 weeks have a higher risk to develop ROP [11, 12].

Additionally, other factors have been implicated in the pathogenesis of ROP such as sepsis, anemia, and chronic lung disease [13]. In our series, anemia represented the third most common risk factor for developing ROP. This can be explained by the fact that the blood used in blood transfusion is rich in adult hemoglobin which binds less firmly to oxygen and releases it to the retinal tissue [14].

Interestingly, Darlow et al. observed that male sex is a significant risk factor, though our data showed an insignificant relationship [15].

Recently, postnatal weight gain, insulin-like growth factor 1 (IGF-1), hyperglycemia, maternal age, and genetic predisposition have also been implicated [16, 17], as well as vitamin E and light exposure [18].

Although initial treatment for ROP was cryotherapy, the current treatment of choice is peripheral diode laser photocoagulation as it appears to be very effective in regressing ROP in agreement with Coats et al. Our cases that needed laser therapy improved and a recession in ROP was noticed in the following examinations.

Furthermore, Parvaresh et al. described the use of transscleral diode laser for threshold ROP.

As VEGF has been identified as one of the most important angiogenic factors in ROP, treatment has been focused on the use of intravitreal anti-VEGF antibodies and several reports exist that describe the use of an off-label anti-VEGF antibody (bevacizumab) [19].

In conclusion, our findings suggest that the development of ROP is strongly associated with early gestational age, low birth weight, and prolonged oxygen exposure. Early retinal screening and regular monitoring are essential and could minimize the possibility of a poor outcome. Constant cooperation between physicians and nursing staff is required in order to avoid undetected cases and further prevent ROP related blindness.

## Figures and Tables

**Table 1:** Association of retinopathy stages with prematurity.

	≤27 weeks with ROP (*e* = 12)	>27 weeks with ROP (*e* = 6)
Stage 1 ROP	0	2
Stage 2 ROP	1	4
Stage 3 ROP		
(I) Mild	1	0
(II) Moderate	2	
(III) Severe	8	

ROP: retinopathy of prematurity;e: eyes.

**Table 2:** Characteristics of premature infants.

Cases	Gestational age (weeks)	Weight (grams)	Gender	Oxygen saturation Sat0_2 _(%) intubated	Intubation period (days)	Cases required blood transfusion(s)	Major complication(s)
I	25	660	Male	92%	138	Yes	RDS
II	27	1150	Male	90%	7	No	RDS, sepsis
III	27	800	Male	93%	14	Yes	RDS
IV	24	850	Female	92%	42	Yes	RDS
V	30	1040	Female	98,1%	2	No	RDS
VI	25	810	Male	89%	38	Yes	RDS
VII	29	1360	Female	84,6%	1	Yes	RDS
VIII	28	970	Female	95,6%	49	Yes	RDS
IX	27	840	Female	93%	28	Yes	RDS, sepsis

RDS: respiratory distress syndrome.
