# Corrigendum to “The Severity of Retinopathy in the Extremely Premature Infants”

**DOI:** 10.1155/2017/4713420

**Published:** 2017-11-22

**Authors:** Alexandra Trivli, Maria Polychronaki, Charoula Matalliotaki, Michail Papadimas, Athina E. Patelarou, Niki Dermitzaki, Michail Matalliotakis

**Affiliations:** ^1^Department of Ophthalmology, Konstantopouleio General Hospital, Athens, Greece; ^2^Neonatology Department, Venizeleio General Hospital of Heraklion, Crete, Greece; ^3^Department of Obstetrics and Gynecology, Venizeleio General Hospital of Heraklion, Crete, Greece; ^4^Department of Anesthesiology, University Hospital of Heraklion, Crete, Greece

In the article titled “The Severity of Retinopathy in the Extremely Premature Infants” [1], affiliations two and three were reversed. The corrected authors' list and affiliations are shown above.
